# Comparison of Indocyanine Green Angiography and Swept-Source Wide-Field Optical Coherence Tomography Angiography in Posterior Uveitis

**DOI:** 10.3389/fmed.2022.853315

**Published:** 2022-05-02

**Authors:** Meng Tian, Guodong Zeng, Christoph Tappeiner, Martin S. Zinkernagel, Sebastian Wolf, Marion R. Munk

**Affiliations:** ^1^Beijing Tongren Eye Center, Beijing Tongren Hospital, Capital Medical University, Beijing, China; ^2^Department of Ophthalmology, Inselspital, Bern University Hospital, University of Bern, Bern, Switzerland; ^3^Bern Photographic Reading Center, Inselspital, Bern University Hospital, University of Bern, Bern, Switzerland; ^4^SITEM Center for Translational Medicine and Biomedical Entrepreneurship, University of Bern, Bern, Switzerland; ^5^Department of Ophthalmology, University Hospital Essen, University Duisburg-Essen, Essen, Germany; ^6^Pallas Klinik, Olten, Switzerland

**Keywords:** OCT angiography (OCTA), indocyanine green (ICG), wide field, uveitis, posterior uveitis, imaging, choriocapillaris (CC), choroid

## Abstract

**Purpose:**

To compare indocyanine green angiography (ICGA) and swept-source wide-field optical coherence tomography angiography (SS-OCTA) for the assessment of patients with posterior uveitis.

**Method:**

SS-OCTA montage images of 5 x 12 x 12 mm or 2 x 15 x 9 mm, covering ~70–90 degree of the retina of consecutive patients with posterior uveitis were acquired. The choriocapillaries and choroidal slabs were compared to findings on ICGA.

**Results:**

Sixty-eight eyes of 41 patients were included (mean age 47.2 ± 20.4 years; 58.5% female). In 23 (34%) lesions were visible on OCTA, but not discernable on ICGA. In turn, out of the 45 eyes with clearly discernable lesions on ICGA, 22 (49%) and 21 (47%) eyes showed no corresponding areas of flow deficit on OCTA in the CC and choroidal slab, respectively. Lesion size strongly correlated among ICGA and OCTA choriocapillaries- (CC) (r = 0.99, *p* ≤ 0.0001) and choroidal slabs (r = 0.99, *p* ≤ 0.0001), respectively. The mean lesion size on the late frames of ICGA (8.45 ± 5.47 mm^2^) was larger compared to the lesion size on OCTA CC scan (7.98 ± 5.47 mm^2^, *p* ≤ 0.0001) and choroidal scan (7.69 ± 5.10 mm^2^, *p* = 0.002), respectively. The lesion size on OCTA CC scan was significantly larger than on the OCTA choroidal scan (*p* ≤ 0.0001).

**Conclusion:**

SS-wide field OCTA may be a promising tool to assess posterior uveitis patients and may replace ICGA to a certain extent in the future.

## Introduction

Posterior uveitis, which is also termed choroiditis, is an inflammation of the choroid and its adjacent tissues (1). Posterior uveitis is associated with vascular changes, usually including retinal and/or choroidal circulation disorders. It is the most infrequent form of uveitis, is quite challenging to diagnose and manage, and can cause vision loss if not treated promptly or adequately.

In clinical routine, fluorescein angiography (FA) is a valuable diagnostic instrument for posterior uveitis as it can help to illustrate the inflammatory process and the anatomical changes in the retina, as well as to assess the severity of inflammation in the posterior segment of the eye. However, FA does not generally visualize the choroidal circulation because the unbound fluorescein molecules leak from the choriocapillaris and mask the choroidal circulation. Indocyanine green angiography (ICGA) in turn uses a tricarbocyanine dye, which is more protein bound than the dye used in FA, reducing the leakage through the fenestrated choriocapillaries and enabling the visualization of the choroidal circulation (2). ICGA is extremely important to assess pathologies involving the choriocapillaris (CC) and the choroidal vasculature in chorioretinal inflammatory diseases (3). Nevertheless, FA and ICGA have some disadvantages: Due to the low resolution of ICGA, the CC can barely be assessed (4). Intravenous dye injection is necessary, which carries the risk of nausea, urticaria, pyrexia, thrombophlebitis and anaphylactic shock. The dye leakage and staining also blur the boundaries of capillary dropout or neovascularization. Most importantly, these techniques are limited in that they can only visualize larger vessels and in case of FA the superficial capillary plexus. However, many clinical and histopathological studies have suggested an association between retinal diseases and choroidal circulation and emphasized the importance of *in vivo* imaging of the CC (5, 6).

Optical Coherence Tomography Angiography (OCTA) is a rapid, non-invasive imaging technique that uses motion contrast imaging with high-resolution volumetric blood flow information to produce angiographic images in seconds. OCTA provides depth resolved images of retinal superficial and deep capillary plexus (SCP, DCP), CC, and deeper choroidal blood flow. OCTA has shown its advantage in the evaluation and management of retinal vascular diseases (7, 8). OCTA allows better assessment of CC, which is displayed as dense and evenly spaced textures of capillaries distributed on a normal OCTA scan (9–11).

In the current study, we aim to assess the potential utility of wide field SS-OCTA in comparison to ICGA in posterior uveitis.

## Materials and Methods

This prospective cross-sectional study was conducted at the Department of Ophthalmology of Inselspital, University of Bern. We analyzed consecutive patients diagnosed with posterior uveitis between October 2017 and July 2020. This study adhered to the tenets of the Declaration of Helsinki and was approved by the ethics committee of Inselspital (ClinicalTrials.gov registration No. NCT02811536).

Adult patients who were diagnosed with posterior uveitis were enrolled. The posterior uveitis diagnosis criteria and disease activity assessment was according to the Standardization in Uveitis Nomenclature (SUN) (12) system and the National Eye Institute (NEI) (13) system. Subjects with any ocular disorders (e.g., corneal opacities, cataract, or dense vitreous hemorrhage/opacities, or with any contraindication to pupil dilation) that impeded high-quality image acquisition were excluded. Patients with a history of eye trauma or other retinal diseases (e.g., diabetic retinopathy, hypertensive retinopathy, central serous chorioretinopathy, macular degeneration) and disorders of the optic nerve (e.g., glaucoma, optic neuropathy) were also excluded.

Ophthalmic examinations were performed in all the patients, including Snellen best-corrected visual acuity (BCVA), slit-lamp examination, intraocular pressure measurements and dilated fundus examination. FA and ICGA were captured with the Spectralis HRA+OCT device (Heidelberg Engineering, Heidelberg, Germany) using the 102° wide field lens with peripheral sweeps in the mid and late phase capturing approximately 150° field of view. The most recent ICGA images were compared with the OCTA images in case ICGA and OCTA were not performed on the same day. If the ICGA had been performed more than 6 weeks before the OCTA or there was a change in disease activity or medication during this period, the patient was excluded.

### OCTA Imaging Acquisition

12 x 12 mm and 15 x 9 mm scans and the wide-field montage OCTA scans consisting of five 12 x 12 mm scans or of two 15 x 9 mm scans, covering about 70 (2 x 15 x 9 mm)-90 (5 x 12 x 12 mm) degrees of posterior pole, were performed and generated using the Swept-source (SS)-OCT (PLEX Elite 9000; Carl Zeiss Meditec, Inc, Dublin, CA). The inbuilt segmentation algorithms were used to create the en-face SS-OCTA wide field slabs. The CC slab segmentation extended from 29 microns to 49 microns beneath the RPE and the choroidal slab segmentation extended from 64 microns to 115 microns below Bruch's membrane (14). Prior creating the montage scans, each individual 12 x 12 and 15 x 9 scan was checked by two trained ophthalmologists and OCTA experts (M.T. and M.R.M.) for alignment errors and artifacts. Minor alignment errors were manually corrected using the inbuilt software. In case of severe artifacts and alignment errors the patient was excluded.

CC and choroidal slabs of the wide-field montage OCTA images were examined for the presence of non-detectable flow signal and flow deficit (15). In order to allow an accurate quantitative lesion size comparison between OCTA and ICGA, respectively, only non-detectable flow signal areas of ≥1/4 optic nerve head (ONH) area of were considered and assessed (16).

### Comparison ICGA and OCTA

**Figure 1:**
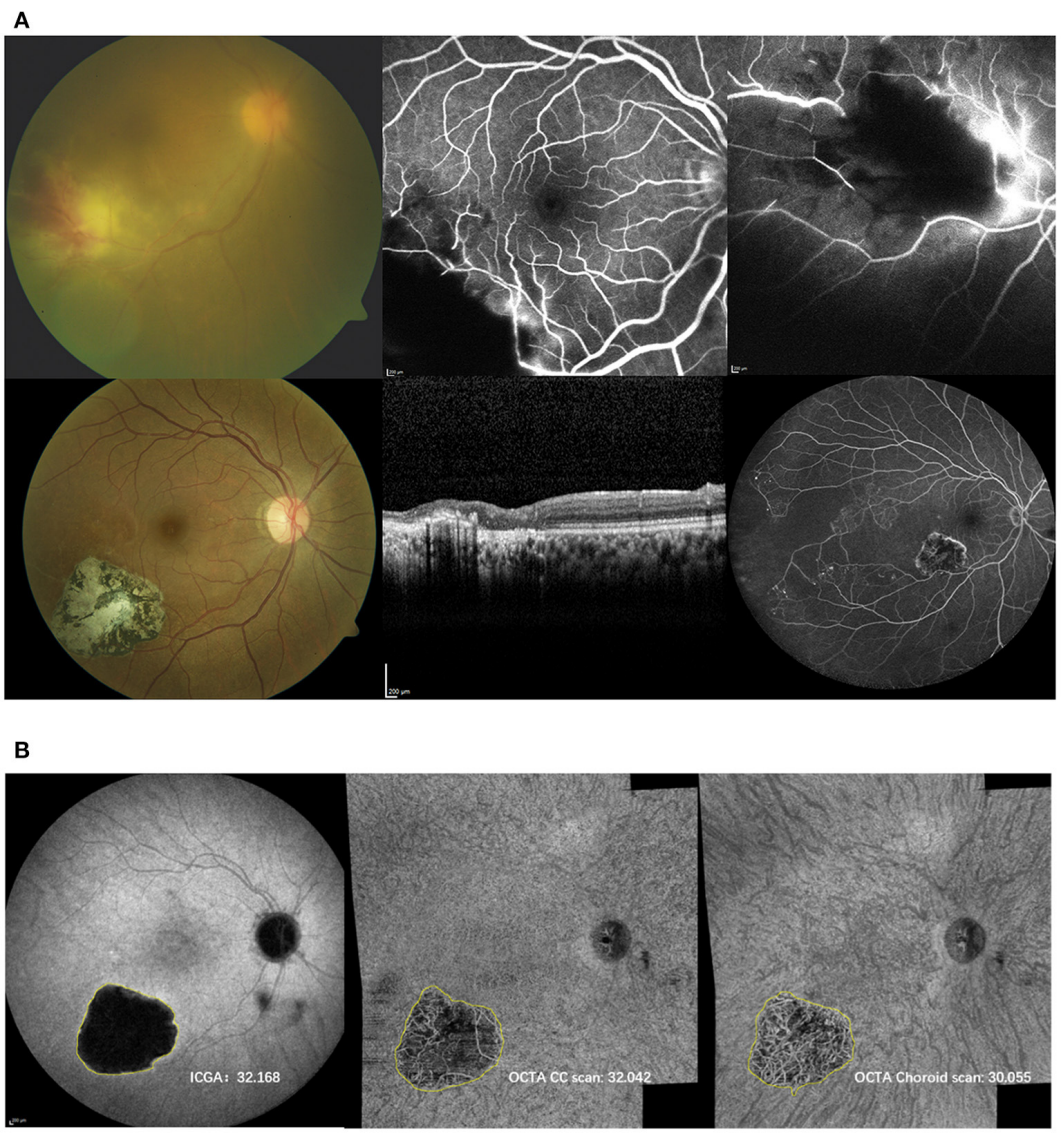
**(A)** Top: Initial presentation of the right eye of a 27-year-old woman with panuveitis OU (DD toxoplamosis retinochoroidtis). Color fundus (CF) image (top left) reveals localized retinochoroiditis with hemorage and vasculitis. Fluorescein angiography (FA) (top middle and right) shows blockage, non-perfusion and vasculitis. Bottom: Follow up: CF (left), optical coherence tomography (middle) and FA acquired at the same time when ICG and OCTA shown in **(B)** were performed. CF depicts inactive well circumscribed chorioretinal scar with pigment migration. On OCT the scar is accompanied with inner and outer retinal atrophy. Wide field FA exhibits the inactive scar with extensive areas of non-perfusion exceeding the scarred area. **(B)** Indocyanine angiography (ICGA) (left) and OCTA choriocapillaris (CC, middle) and choroidal (right) scans of respective patient including the measurements of the lesion (mm^2^). The lesion appeared larger on ICG than on the OCTA CC and choroidal slabs.

Late frame images of ICGA were examined for the presence of hypofluorescenct lesions, while CC and choroidal OCTA slabs were examined for the presence of flow deficit and non-detectable flow signal, respectively. The size of the lesions visible on the wide-field OCTA images of the CC and choroidal slabs was compared to the size of the hypofluorescent-lesions on ICGA in late phase (≥15 min). Affine transformation was used for registration between the different imaging modalities. Specifically, blood vessel intersection points were manually selected from the ICGA and OCTA retinal slab to define an affine transformation matrix, which was then applied to OCTA CC scan and choroidal scan for registration (Figure 1). ImageJ software (Version1.8.0, National Institutes of Health, Bethesda, Maryland, USA) was used to measure the area of each individual lesion on a selected OCTA and ICGA image. The boundaries were drawn manually on the same image by 2 independent graders. When discrepancies in lesion area between graders were beyond 50% or 2 mm^2^, an open adjudication between the 2 graders was performed, and the area was redrawn or one of the previous drawings was accepted as the final measurement. Otherwise, the average of the areas determined by the 2 graders was used for the area assessment.

Vascular network abnormalities on OCTA were then compared to ICGA.

### Statistical Analysis

Statistical analysis of the data was performed using commercial software (SPSS version 23.0, Inc, Armonk, NY). Descriptive statistics (percentages, means and standard deviation) were computed for demographic and clinical variables. Chi-squared test was used for qualitative data analysis. Paired *T*-test was used to analyze the quantitative data. Pearson test was used for the correlation of quantitative data. Intraclass correlation coefficients (ICCs) was used to assess intergrader reproducibility. A value of *P* ≤ 0.05 was considered statistically significant.

## Results

A total of 90 eyes from 58 patients were screened. Out of these, 68 eyes of 41 patients were enrolled (mean age 47.2 ± 20.4 years). Twenty-four patients (58.5%) were female. The other eyes had to be excluded as the time frame between OCTA and ICGA exceeded 6 weeks or there was a change in medication and disease activity during this period or OCTA scans showed severe artifacts, which didn't allow an accurate comparison with ICGA. Baseline clinical characteristics and demographics are summarized in Table 1. The majority of eyes suffered from idiopathic posterior uveitis (57.4%). The second most frequent underlying diagnose was Birdshot chorioretinitis (16.2%), followed by VKH chorioretinitis (8.8%) and ocular sarcoidosis (5.9%). Other underlying diagnosis included presumed ocular tuberculosis, Behet, serpiginous chorioretinopathy and APMPPE (2.9% each). The mean Snellen BCVA was 0.75 ± 0.33. 18 eyes (26.5%) exhibited clinically active inflammation based upon SUN and NEI criteria. The remaining eyes were classified as inactive. The mean disease duration was 9.5 ± 5.6 months (median: 11 month, range: 3–15 months). Most patients [*n* = 23 (56%), 36 eyes (52.9%)] were on systemic immune modulating therapy. Ten eyes (14.7%) received topical treatment and the remaining eyes had no treatment.

**Table 1:** Baseline clinical characteristics of included patients and eyes.

Age (years)	47.2 ± 20.4
**Sex**	*n* (41 patients)
Male	17 (41.5%)
Female	24 (58.5%)
**Laterality**	*n* (41 patients)
Unilateral	14 (34.2%)
Bilateral	27 (65.8%)
**Uveitis disease activity**	*n* (68 eyes)
Active	18 (26.5%)
Inactive	50 (73.5%)
**Main affected anatomical site**	*n* (68 eyes)
Choroiditis	15 (22.1%)
Retinitis	17 (25%)
Retinochoroiditis	36 (52.9%)
Best corrected visual acuity (Snellen)	0.75 ± 0.33
**Treatment**	*n* (68 eyes)
No medication	22 (32.4%)
Systemic immune modulating therapy (IMT)	36 (52.9%)
Topical only	10 (14.7%)
**Diagnosis**	*n* (68 eyes)
Ocular sarcoidosis	4 (5.9%)
Presumed ocular tuberculosis	2 (2.9%)
Behcet disease associated posterior uveitis	2 (2.9%)
Vogt–Koyanagi–Harada choroiditis	6 (8.8%)
Birdshot chorioretinitis	11 (16.2%)
Serpiginous choroidopathy	2 (2.9%)
APMPPE	2 (2.9%)
Idiopathic posterior uveitis	39 (57.4%)
**SS-OCT findings**	*n* (68 eyes)
Epiretinal membrane	19 (27.9%)
Cystoid macular edema	21 (30.9%)

### Lesion Presence: OCTA Compared to ICGA

Out of the included 68 eyes, 45 (66%) presented with clearly discernable hypofluorescent lesions in the late frames of the ICGA. The remaining 23 (34%) eyes had lesions visible on OCTA, but no lesions were discernable on ICGA. In turn, out of the 45 eyes with clearly discernable lesions on ICGA, 22 (49%) and 21 (47%) eyes showed no corresponding areas of flow deficit on OCTA in the CC and choroidal slab, respectively. In 14 of these 22 eyes (64%) the lesions were outside of the captured area of the OCTA image. This translates to 8 (36%) and 7 (32%) eyes, where lesions should have been visible on OCTA, but were only appreciable on ICGA. Details are shown in Table 2.

**Table 2:** Cross table presenting the prevalence of non-perfusion visible on ICGA and OCTA.

		**OCTA CC (n)**		**Total (*n*)**
		**Lesions present**	**Lesions absent**	
ICGA n	Lesions present	23	22	45
	Lesions absent	5	18	23
Total (n)		28	40	68
		**OCTA Choroid (n)**		**Total (** ***n*** **)**
		**Lesions present**	**Lesions absent**	
ICGA n	Lesions present	24	21	45
	Lesions absent	6	17	23
Total (n)		30	38	68

### Comparison of Lesion Size Between OCTA and ICGA

**Figure 2:**
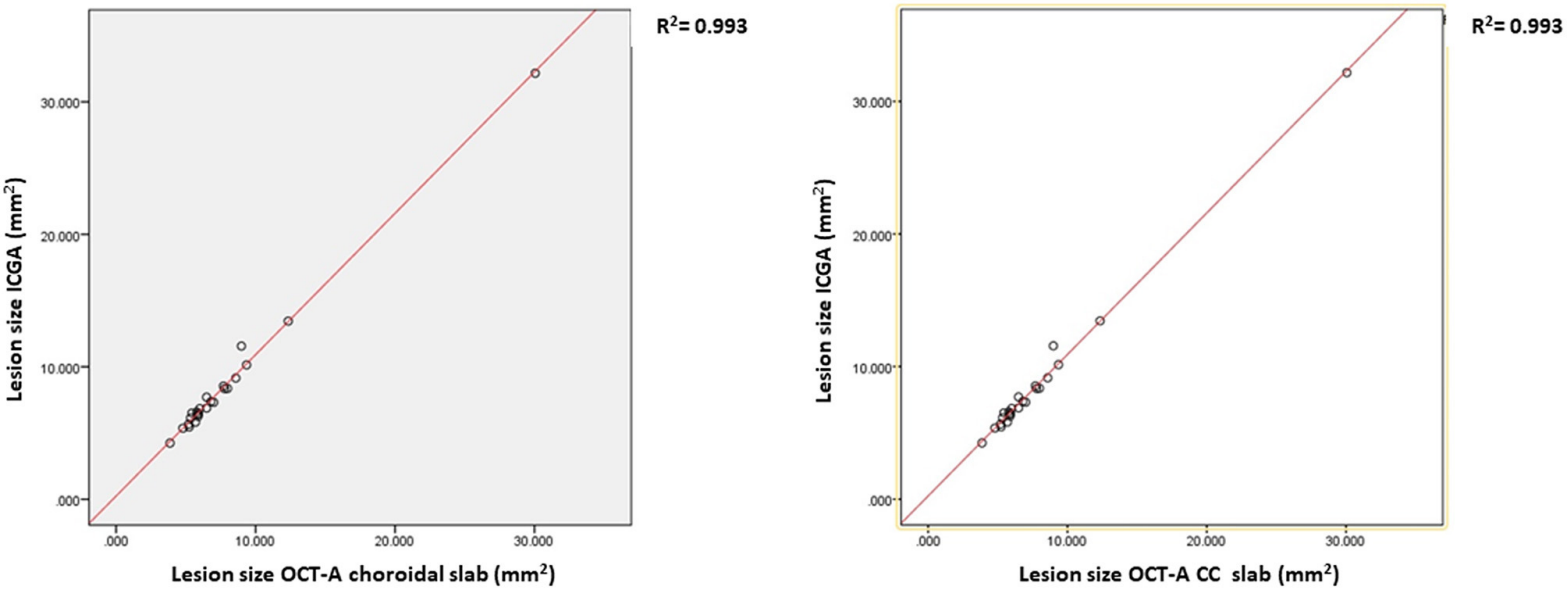
The correlation and the regression lines between the lesion size on Indocyanine angiography (ICGA) and OCTA choriocapillaries (CC) slabs **(left)** and between the ICGA and the OCTA choroidal slabs **(right)**.

In 24 (for the choroidal slab) and in 23 (for the CC slab) cases, the lesions were clearly visible and fully captured on ICGA and OCTA, respectively. In these cases, the lesion size was compared among ICGA and wide field OCTA CC scans and choroidal scans, respectively. ICCs for lesion size assessment on ICGA images was 0.98 (95% confidence interval, 0.98–0.99). For en-face OCTA CC and choroidal slabs, it was 0.94 (95% confidence interval, 0.94–0.97) and 0.95 (95% confidence interval, 0.93–0.98), respectively. The mean lesion size on ICGA (8.45 ± 5.47 mm^2^) was larger compared to the lesion size on OCTA CC scan (7.98 ± 5.47 mm^2^, *p* ≤ 0.0001) and choroidal scan (7.69 ± 5.10 mm^2^, *p* = 0.002), respectively (Figure 1). The lesion size on OCTA CC scans was significantly larger than on the OCTA choroid scans (*p* ≤ 0.0001). The lesion size on ICGA strongly correlated with the size on the OCTA CC scan (r = 0.99, *p* ≤ 0.0001) and choroidal scan (r = 0.99, *p* ≤ 0.0001), respectively (Figure 2).

## Discussion

The aim of this study was to assess the utility of OCTA in patients with posterior uveitis compared to the gold standard image modality ICGA. We used the affine transformation for registration between ICGA and OCTA and compared the lesion size among ICGA, OCTA CC and choroidal scans.

In our series, the presence and size of capillary non-perfusion on the wide-field montaged OCTA CC scans and choroidal scans correlated with the hypofluorescent-lesions on the late frames of ICGA. This is consistent with previous studies. Pakzad-Vaezi et al. (17) reported that the CC flow voids in patients with serpiginous choroiditis were highly correlated with the ICGA lesions and grew in size with the onset of inflammation and reduced in size after treatment. Another study of 18 eyes with tubercular serpiginous-like choroiditis also found a well correlated morphology between OCTA and ICGA (18). Klufas et al. (19) assessed patients with acute posterior multifocal placoid pigment epitheliopathy (APMPPE) and found that the CC flow reduction corresponded well with the ischemic lesions on ICGA but were larger on OCTA. These lesions significantly improved and decreased in size during follow up. Also, in patients with multifocal choroiditis, OCTA showed flow-void areas at the level of choriocapillaris that corresponded to the hypofluorescent areas on ICGA (20). In a recent study, the appearance of granulomatous lesions were assessed on 12 x 12 mm SS-OCTA slabs and compared to ICGA. In over 94% of cases hypofluorescent lesions on ICGA had corresponding areas of flow voids on OCTA (21). However, several areas of flow voids were detected on OCTA which were invisible on ICGA. Also, in our study some lesions were visible on ICGA but invisible on OCTA and vice versa. Our results expand the range of assessable diseases and the field of view (FOV) using montage wide field OCTA and highlight that OCTA could be a promising tool for the evaluation of the choroidal vascular changes in posterior uveitis. The main limitation of OCTA is its (yet) limited field of view. Wide field ICGA images in this study were performed with the Spectralis 102° wide field lens and peripheral sweeps, leading to a FOV of approximately 150°, while montage wide field OCTA captured a max area of approximately 90°FOV. It is therefore not surprising that the limited FOV of OCTA imaging was the main reason (64%) why no corresponding area of flow deficit was visible on OCTA compared to ICGA. Lesions localized within 70–90° FOV, resulted in comparable sensitivity; with 8 cases having lesions visible on ICGA but not detectable on OCTA, and with 5 cases having lesions detectable on OCTA but being missed on ICGA. The limited FOV as major limitation may be transient though. SS-OCT and SS-OCTA modules like the Xephilio OCT-S1 from Canon are already commercially available, which allow single 20 x 23 mm OCTA scans. These scans can be montaged, and ultrawide field images, capturing the anterior edge of all 4 vortex veins can be produced (22).

In our series, the lesion size on OCTA CC scan was significantly larger than on the choroidal scan. In posterior uveitis the choriocapillaris is most of the time the primary and more severely affected layer, which may be the underlying reason for this finding. The here included patients were diagnosed among other underlying diseases with APMPPE, serpiginous choroiditis, Vogt–Koyanagi–Harada (VKH), sarcoidosis and tuberculosis. A previous study on eyes with APMPPE and related placoid disorders revealed that the inner choroid is considered to be the leading site in the pathogenesis of the disease and is associated with the secondary destruction of photoreceptors (19). Although active APMPPE causes perfusion deficits in the CC and the choroid, the choroidal deficits resolve much faster (23). This reinforces that the CC is the primarily affected layer in this disease. Also, in serpiginous choroidopathy, the CC seems to be the primary location of inflammation, leading to largest lesions at the level of the CC, especially in active disease (17). Even in choroidal, granulomatous diseases such as sarcoidosis, tuberculosis and Vogt–Koyanagi–Harada flow deficits may appear larger in the CC than in the choroid, as the underlying choroidal granulomas can cause ischemia in the CC.

Interestingly the lesions appeared slightly larger on ICGA than on the OCTA CC and choroidal slabs. Previous studies have already demonstrated that the lesion size varies on different image modalities (17, 24). The underlying reason however is yet unclear. Chang et al. (25) investigated the uptake properties of ICGA by the RPE and found that ICGA is normally absorbed by the RPE, resulting in physiological background hyperfluorescence. However, in some retinal diseases, like in posterior uveitis, absorption of ICGA is disturbed, which may explain the larger size of lesions on ICGA lesions compared to OCTA CC and choroidal scans. Considering that neither the cause of hypofluorescence on ICGA nor the cause of flow deficit on OCTA in chorioretinal inflammatory diseases is fully understood yet, it makes it even harder to discuss and understand their potential differences. The hypofluorescence on ICGA may be induced by choroidal hypo- or non-perfusion or by an impaired filling and staining of the choroidal tissue due to inflammatory foci (26). On OCTA areas of flow deficit may be caused by vessel displacement due to solid lesions such as granulomas or due to signal attenuation due to completely absent perfusion or by blood flow speed below detection level of the OCTA module. It may be also induced by blockage of signal due to inflammatory cells (27, 28). This also indicates that a lesion appears generally larger in active disease due to the additional blockage of inflammatory cells, while inactive lesions may present smaller, representing areas of flow deficit solely due to real non-perfusion and ischemia. Also, the phase of ICGA has significant impact on the appearance and size of the inflammatory choroidal lesions. A lesion which extends throughout the whole choroid will remain completely hypofluorescent throughout all ICGA frames and may appear larger, while lesions infiltrating only a part of the choroid may become isofluorescent in the very late frames and appear smaller (26). Also, the underlying cause in posterior uveitis has to be considered. APMPPE, for example has its main focus in the choriocapillaries and outer retina, while the main site of inflammation in Birdshot chorioretinopathy is found in the outer choroid, slowly progressing toward the choriocapillaries and the outer retina. Thus, inflammatory choroidal lesions of different underlying entities present differently on ICGA and may also lead to differences in the CC and choroidal OCTA slabs. A recent scientific abstract assessed choriocapillaries flow deficits of different patients with posterior uveitis and explored that the ICGA seemed to reveal larger flow deficits as suggested by OCTA, which is consistent to our finding (29). Interestingly, Pichi et al. (21) compared the size of granulomatous lesions on ICGA and SS-OCTA choroidal slabs and found that lesions on OCTA appeared in mean 0.08 mm2 larger than on ICGA. In his study single 12 x 12 mm OCTA scans were used and only smaller granulomatous lesions of patients with VKH, sarcoidosis and tuberculosis were included, while our study included only lesions of larger sizes and the whole variety of posterior uveitis entities. These differences may explain the divergent findings.

It should be noted that the present study has several limitations. First, we used the affine transformation for registration between different imaging modalities. The low resolution and artifacts of OCTA may cause imprecise registration and lead to an inaccurate result in the measurement. In addition, although we have excluded OCTA images with severe artifacts, almost all images had some sorts of artifacts and/or segmentation errors, which may have impacted the assessment as well. A previous study of our group showed that 100% of montage wide field OCTA images present with artifacts including but not limited to displacement-, shadowing- and artifact and vessel displacement (30). The inbuilt Plex Elite Zeiss segmentation of the CC and choroidal slab was used in this study; customized segmentations, precisely adapted to the underlying conditions may be beneficial. Furthermore, the small number of patients was also one limitation of our study. Finally, we have included a variety of disease entities. Posterior uveitis is a very heterogenous diagnosis, and primary site of inflammation can be either found in the retina, CC or the choroid. The pooling of data of different underlying conditions may limit the interpretability as well. A subanalysis of different underlying conditions would be really interesting but is not feasible here due to the rather small numbers of different underlying pathologies. However, large multicenter trials in uveitis usually include the whole variety of posterior uveitis. This is why we also included a variety of such entities in this study, in order to assess the utility of OCTA in posterior uveitis in general compared to the Gold standard modality ICGA. We have only included lesions ≥1/4 ONH area. This has several reasons. First it helped us to ensure that the areas of flow deficit were caused by inflammatory lesions and not by artifacts or perturbances of blood flow related to inflammation. Secondly, we registered images using affine transformation and the lower resolution of wide field OCTA may cause imprecise registration. In case of very small lesions, we probably would have received inaccurate results. But this limitation also implies, that we have ignored many lesions, which were smaller, such as granulomas found in VKH or sarcoidosis.

## Conclusion

Our study demonstrated that SS-OCTA is a fast, non-invasive and high-resolution choroidal imaging technique to assess choriocapillaries and choroidal lesions in posterior uveitis. It may be useful to monitor patients and may obviate the need for repeated dye-based angiography. Further studies however are warranted to confirm the findings and add additional information soon.

## Data Availability Statement

The original contributions presented in the study are included in the article/supplementary materials, further inquiries can be directed to the corresponding author/s.

## Ethics Statement

The studies involving human participants were reviewed and approved by Bern Ethics Committee. Written informed consent was collected for each patient.

## Author Contributions

MT: data collection, data analysis, and drafting manuscript. GZ: data analysis and revising manuscript. CT: data collection and revising manuscript. MZ and SW: study design, data collection, and revising manuscript. MM: study design, data collection, data analysis, and drafting and revising manuscript. All authors contributed to the article and approved the submitted version.

## Conflict of Interest

The authors declare that the research was conducted in the absence of any commercial or financial relationships that could be construed as a potential conflict of interest.

## Publisher's Note

All claims expressed in this article are solely those of the authors and do not necessarily represent those of their affiliated organizations, or those of the publisher, the editors and the reviewers. Any product that may be evaluated in this article, or claim that may be made by its manufacturer, is not guaranteed or endorsed by the publisher.
