# Corrigendum: Comparison of indocyanine green angiography and swept-source wide-field optical coherence tomography angiography in posterior uveitis

**DOI:** 10.3389/fmed.2024.1426456

**Published:** 2024-05-14

**Authors:** Meng Tian, Guodong Zeng, Christoph Tappeiner, Martin S. Zinkernagel, Sebastian Wolf, Marion R. Munk

**Affiliations:** ^1^Beijing Tongren Eye Center, Beijing Tongren Hospital, Capital Medical University, Beijing, China; ^2^Department of Ophthalmology, Inselspital, Bern University Hospital, University of Bern, Bern, Switzerland; ^3^Bern Photographic Reading Center, Inselspital, Bern University Hospital, University of Bern, Bern, Switzerland; ^4^SITEM Center for Translational Medicine and Biomedical Entrepreneurship, University of Bern, Bern, Switzerland; ^5^Department of Ophthalmology, University Hospital Essen, University Duisburg-Essen, Essen, Germany; ^6^Pallas Klinik, Olten, Switzerland

**Keywords:** OCT angiography (OCTA), indocyanine green (ICG), wide field, uveitis, posterior uveitis, imaging, choriocapillaris (CC), choroid

In the published article, there was an error in affiliation ranking for “Meng Tian.” Instead of the sequence “^1^Department of Ophthalmology, Inselspital, Bern University Hospital, University of Bern, Bern, Switzerland, ^2^Bern Photographic Reading Center, Inselspital, Bern University Hospital, University of Bern, Bern, Switzerland, ^3^Beijing Tongren Eye Center, Beijing Tongren Hospital, Capital Medical University, Beijing, China,” it should be “^1^Beijing Tongren Eye Center, Beijing Tongren Hospital, Capital Medical University, Beijing, China, ^2^Department of Ophthalmology, Inselspital, Bern University Hospital, University of Bern, Bern, Switzerland, ^3^Bern Photographic Reading Center, Inselspital, Bern University Hospital, University of Bern, Bern, Switzerland.”

The authors apologize for this error and state that this does not change the scientific conclusions of the article in any way. The original article has been updated.

